# Consolidation of motor sequence learning eliminates susceptibility of SMAproper to TMS: a combined rTMS and cTBS study

**DOI:** 10.1007/s00221-022-06358-y

**Published:** 2022-04-07

**Authors:** Willem B. Verwey, Benedikt Glinski, Min-Fang Kuo, Mohammad Ali Salehinejad, Michael A. Nitsche

**Affiliations:** 1 Faculty of Behavioural, Management and Social Sciences, Department of Learning, Data-Analytics and Technology, Cognition, Data and Education Section, University of Twente, PO Box 217, 7500 AE Enschede, The Netherlands; 2 Department of Kinesiology, Non-Invasive Brain Stimulation Laboratory, Texas A&M University, College Station, TX USA; 3 Department of Psychology and Neurosciences, Leibniz Research Centre for Working Environment and Human Factors, Dortmund, Germany; 4 Department of Neurology, University Medical Hospital Bergmannsheil, Bochum, Germany

**Keywords:** Consolidation, Transcranial magnetic stimulation, Continuous theta burst stimulation, Discrete sequence production task, Motor sequences

## Abstract

Earlier research suggested that after 210 practice trials, the supplementary motor area (SMA) is involved in executing all responses of familiar 6-key sequences in a discrete sequence production (DSP) task (Verwey, Lammens, and van Honk, 2002). This was indicated by slowing of each response 20 and 25 min after the SMA had been stimulated for 20 min using repetitive transcranial magnetic stimulation (rTMS). The present study used a similar approach to assess the effects of TMS to the more posterior SMAproper at the end of practice and also 24 h later. As expected stimulation of SMAproper with 20 min of 1 Hz rTMS and 40 s of continuous theta burst stimulation (cTBS) immediately after practice slowed sequence execution relative to a sham TMS condition, but stimulation on the day following practice did not cause slowing. This indicates that offline consolidation makes learning robust against stimulation of SMAproper. Execution of all responses in the sequence was disrupted 0, 20, and 40 min after rTMS, but after cTBS, this occurred only after 40 min. The results suggest that it is implicit sequence knowledge that is processed by the SMAproper and that consolidates.

## Introduction

Transcranial magnetic stimulation, or TMS, involves administering short magnetic pulses through the skull to cortical tissue. This influences cortical processing through enhancement or depression of synaptic activity during and after the stimulation process, depending on the stimulation protocol (Di Lazzaro et al. 2011). TMS opens the way for various therapeutic treatments (e.g., Kobayashi and Pascual-Leone, 2003; Takeuchi et al. 2008). It also provides a way of investigating the functions of cortical areas for human behavior, because it can establish a causal link between brain function and behavior (Pascual-Leone et al. 1992; Walsh and Cowey 2000). The present study specifically looked into the contribution of the posterior part of the supplementary motor area (SMA), the SMAproper, to the execution of two familiar 6-key sequences and whether this contribution consolidates over 24 h. This was explored with two inhibitory TMS protocols to examine whether the behavioral effects of the traditional 20 min offline 1 Hz repetitive TMS (*rTMS*) protocol (Pascual-Leone 1999; Pascual‐Leone et al. 1991) can be achieved also with a 40 s offline continuous theta burst stimulation (*cTBS*) protocol (Huang et al. 2005).

### The effects of TMS on the DSP task

The discrete sequence production (DSP) task we used initially involves reacting to two fixed series of, typically, 6 or 7 stimuli (Abrahamse et al. 2013; Verwey 1999). During practice, participants learn to select and execute these keying sequences as if these constitute a single response. This task has been used for over two decades in research on motor sequence learning (Verwey 1996, 1999). Behavioral research with this DSP task resulted in various cognitive models (Abrahamse et al. 2013; Verwey 2001; Verwey et al. 2015). In addition, a variety of DSP studies have explored the neural substrate of motor sequence learning using EEG (e.g., De Kleine and Van der Lubbe 2011; Sobierajewicz et al. 2017), fMRI (Jouen et al. 2013; Verwey et al. 2019), and also TMS (Ruitenberg et al. 2014; Verwey et al. 2002). Those studies confirmed that the SMA is involved in executing practiced DSP sequences.

The first DSP study that assessed the effects of TMS of the SMA involved participants initially learning two fixed series of seven letters (Verwey et al. 2002). The first letter was used later as imperative stimulus for the 6-key sequence that was represented by the ensuing six letters. We used learned letter series, instead of the more typical display of key-specific stimuli, because at the time, these stimuli were suspected to reduce the contribution of the SMA. This conjecture recently received support from the finding that the onset of all key-specific stimuli in the DSP task capture attention and this triggers each response even after extended practice (Verwey et al. 2020). The results of this TMS study confirmed involvement of the SMA in the DSP task in that 20 and 25 min after 20 min of 1 Hz offline TMS of this area all responses of the two familiar 6-key sequences were slowed by 19 ms. Only immediately after rTMS slowing was not observed.

Given the functional distinction between the anterior part of SMA, preSMA, and the posterior part, SMAproper (Hardwick et al. 2013; Shima and Tanji 2000), the Verwey et al. (2002) study was followed by two TMS studies that specifically stimulated preSMA (Kennerley et al. 2004; Ruitenberg et al. 2014). Those studies were carried out, because it was shown that familiar discrete motor sequences that include more than 4 or 5 responses are increasingly executed as two successive segments (or ‘chunks’) of which the first response is relatively slow (Bo and Seidler 2009; Verwey et al. 2009; Verwey and Eikelboom 2003). Stimulating the preSMA appeared to slow only the first response of both segments and not the other responses. This led to the conclusion that in the case of familiar DSP sequences, the preSMA initiates each segment. However, this also suggests that the effect of SMA stimulation on each response in the sequence (Verwey et al. 2002) was actually caused by stimulation of SMAproper.

Stimulation of the preSMA in Ruitenberg et al. (2014) and of the SMA in Verwey et al. (2002) occurred at locations only 1.5 cm apart, that is, 5.5 and 4 cm anterior of the Cz location, respectively. However, TMS studies are known to often produce variable results (de Jesus et al. 2014; Klomjai et al. 2015; Lang et al. 2006), possibly also because of individual differences in cortical physiology (Latorre et al. 2019; Maeda et al. 2000). We, therefore considered it important to show that all responses in a practiced DSP sequences are indeed slowed when TMS is especially targeting SMAproper (i.e., 3 cm anterior of Cz, Lefaucheur et al., 2020).

### Consolidation

Offline consolidation is the phenomenon that memory traces that are initially labile in that they are susceptible to subsequently executed tasks and brain stimulation, become robust to this interference during the 4–8 h that follow practice. Offline consolidation sometimes also enhances skill (Brashers-Krug et al. 1996; Handa et al. 2016; Robertson et al. 2004; Walker et al. 2003), and for motor sequences, this enhancement may develop across a period as long as 72 h (Kim et al. 2016; Wright and Kim 2019). Consolidation has been shown to contribute to the well-known benefit of random over blocked practice in the contextual interference paradigm (Kantak et al. 2010; Kim and Wright 2020; Lin et al. 2011; Verwey et al. 2021). This benefit of random practice is most likely due to the repeated preparation of motor sequences during random practice which does not occur with short sequences in the blocked practice regime (Verwey et al., 2021).

Verwey et al. (2002) used a within-subject design to prevent short-term effects of TMS on the subsequent sham condition by administering real and sham stimulation on successive days. In retrospect, offline consolidation may have eliminated the effect of TMS in the participants who were stimulated on the second day and averaging across groups with different stimulation order obscured this. We, therefore, tested whether response slowing by TMS of SMAproper would be reduced on the day after practice.

### rTMS and cTBS

The earlier TMS study involved the classic offline rTMS protocol (Verwey et al. 2002). This protocol involves administering for 20 min brief magnetic pulses at 1 Hz (Hoogendam et al. 2010; Pascual-Leone 1999; Pascual‐Leone et al. 1991). It is called an offline procedure, because it is administered before the participant carries out the task of interest. A more recently developed protocol is offline cTBS (Casula et al. 2014; Dafotakis et al. 2008; He et al. 2020; Strzalkowski et al. 2019; Zafar et al. 2008). This protocol involves only 40 s stimulation during which 5 Hz bursts of magnetic pulses are administered (Huang et al. 2005). Obviously, researchers prefer the short duration cTBS over the longer lasting rTMS if they know that these protocols have the same effects, also because such a short duration simplifies TMS coil fixation. Yet, little is known about the behavioral differences between these stimulation protocols in motor sequencing studies. We, therefore, investigated in the present study whether the inhibitory effect of 40 s offline cTBS is comparable with that following 20 min offline rTMS. Given that Verwey et al. (2002) found effects of rTMS only after 20 and 25 min and not immediately after stimulation, and that after-effects of TMS have been found to last up to 1.5 h (Hamada et al. 2008), we compared the effects of rTMS and cTBS on the discrete keying sequences 0, 20, and 40 min after completion of both stimulation protocols.

### The present experiment

Participants practiced two 6-key DSP sequences for 210 trials per sequence after they had learned verbal sequences consisting of 1 stimulus letter and 6 key-specific letters, just like in Verwey et al. (2002). Yet, this time we placed the TMS coil 3 cm, instead of 4 cm, anterior of Cz to specifically target SMAproper and to reduce possible stimulation of preSMA. We explored whether TMS of the SMAproper immediately after practice would be more inhibiting than TMS administered 24 h later to examine whether offline consolidation protects memory against the disruptive effect of TMS. We further assessed whether TMS targeting SMAproper would slow all key presses of practiced DSP sequences, whether the disrupting effect of 20 min rTMS could be obtained also by the more efficient 40 s cTBS, and whether the inhibitory effect develops in the same way for both stimulation protocols by assessing the TMS effect 0, 20, and 40 min after the end of stimulation. We tested awareness of the sequences at the end of the experiment to explore whether the SMAproper is involved in the application of implicit or explicit sequence knowledge.

## Methods

### Participants

The sample comprised 32 participants (24 females) in the age range of 18–34 years (*M* = 25.0, SD = 3.6). The participants were recruited via social media advertisements and were monetarily compensated for taking part in the study or awarded with study credits. All participants were right-handed, and had normal or corrected to normal visual acuity. Medical examination revealed a good physical and mental health condition for all participants. The medical examinations included a standard pre-screening questionnaire concerning presence and history of diseases, and presence of exclusion criteria for TMS, assessment of blood pressure, and a neurological examination of coordination, vision, sensory and motor skills. Alcohol, nicotine and drug addiction and the intake of medication affecting the central nervous system led to exclusion. In line with general TMS safety guidelines (Rossi et al. 2009), participants were asked whether they had been diagnosed with chronic or residual neurological diseases, epilepsy (or prior evidence of epileptic seizures), skull fractures or brain tissue lesions, intracerebral ischemia or bleeding and local or global aphasia, and whether they had implanted pacemakers or deep brain stimulation. Eventually, none of the participants was excluded from the analysis. The study was approved by the IfADo ethics committee (proposal number 2020–172). The research conformed to the Declaration of Helsinki guidelines. Written informed consent was obtained from all participants before participation.

### Apparatus

Stimulus presentation and response registration were controlled by E-prime© 2.0 that was installed on a computer running Windows 7. The computer was disconnected from the internet, and most background applications were disabled. The keying sequences were pressed on a standard QWERTZ-keyboard with a fast PS2 connection. The stimuli were presented on an Iiyama HM703UT tube monitor with a screen diagonal of 43 cm.

rTMS and cTBS were delivered using a Mag and More PowerMAG Clinical pp TMS device with a 70 mm figure-of-eight coil. The coil was statically placed on the participants’ head using a Mag and More coil holder. The head of the participant was fixated utilizing a vacuum pillow.

### Design

We assessed effects of stimulation on response times (RTs) and error rate using a mixed, single-blinded, sham-controlled research design^1^. One participant group received a 20 min 1-Hz rTMS intervention and the other group a 40 s 50-Hz cTBS intervention. Participants in both groups received both real and sham stimulation. The real and sham stimulation sessions took place on consecutive days with 24 h between sessions and their order was counterbalanced across the participants. All participants were randomly assigned to one of the groups and were kept blind to the type of stimulation (cTBS/rTMS) and whether stimulation was real or a sham. On Days 1 and 2, every participant performed a baseline block before, and three test blocks 0, 20 and 40 min after, stimulation.

### Behavioral tasks

The experiment employed the same discrete sequence production (DSP) task as in Verwey et al. (2002). It involved participants pressing with the left hand two 6-key sequences in response to a single sequence-specific stimulus. To that end, participants first learned two 7-letter series. These series started with a sequence-specific stimulus (one of the letters O, X, E, D, G, I, L, or M) followed by a series of 6 letters representing the 6 keys to be pressed (consisting of the letters C, V, B, and N, e.g., ONCBNCB). One sequence involved a 2 × 3, the other a 1 × 6 sequence. The 2 × 3 sequence consisted of a 3-key segment that was repeated. It was one of a set of 4 alternative sequences (NCBNCB, CVNCVN, VBCVBC or BNVBNV). The 1 × 6 sequence did not involve such a repetition and was one of 4 alternatives too (BCVNVC, NVBCBV, CBNVNB or VNCBCN). The 2 × 3 and 1 × 6 sequences always started with another key for each participant.

During practice and test blocks, the participants executed their two 6-key sequences in response to the sequence-specific stimulus. Sequence completion and error messages were followed by a 1500 ms interval before the next sequence-specific stimulus was presented. Each trial consisted of executing an entire sequence. Practice involved three 140-trial blocks, yielding a total of 210 practice trials per sequence. The four test blocks included 40 trials each. The participants were urged in the general instruction at the start of the experiment and by the RT and error percentage feedback at the end of each trial block to always stay below 8% error rate. The 8% itself was arbitrary but seemed a reasonable percentage to obtain few errors and a reasonably fast execution of the keying sequences.

After the end of the last test block on day two, the participants performed a computerized awareness task that included two tests that were administered in a counterbalanced order (Verwey and Dronkers 2019). Both tests involved clicking with the mouse, in a self-chosen sequence order, six successive element-specific squares on the display in the order participants thought they had pressed keys. During the spatial awareness test, the mentioned elements were displayed as four-square placeholders lined up next to each other, just like in the practice and test blocks. The participants were asked to click the two sequences that they had learned and executed throughout the experiment in the same succession with the computer mouse. Each placeholder was empty and represented one key on the keyboard (c, v, b, and n). This test examined explicit knowledge of the locations of the successively pressed keys, that is, explicit spatial sequence knowledge. During the verbal awareness test, four placeholders were displayed at the top, left, bottom and right across the screen in a rhombus shape. This time, each placeholder contained one of the letters of the two sequences (c, v, b, n). The participants were again asked to click the placeholder based on the succession of the two learned sequences. This test examined explicit verbal knowledge of the order of the stimulus letters. Finally, participants were asked to indicate whether reproducing the sequences in this awareness task had involved either (a) recalling the successive letters of the key pressed, (b) recalling the locations of the stimuli and or keys pressed, or reconstructing the sequence by either (c) tapping the sequence on the table top or (d) in the mind. The final option was that (e) they had no idea. They filled this in separately for the spatial awareness test and the verbal awareness test, and were then asked how certain they had been of their answers. Finally, they were told about the sham and real stimulation and indicated whether they thought that the real stimulation had taken place on the first or second day or whether they had no idea. During the awareness task, the keyboard was covered to ensure that the participants relied on memory recall instead of recognition.

### TMS

For rTMS and cTBS of SMA the center of the figure-of-eight coil was positioned 3 cm anterior to Cz, according to the international 10–20 system of electrode placement. For the control condition, a sham coil was used which produced the same sound as the normal coil but no magnetic pulse. The junction area of the coil was positioned with the handle pointing backwards and parallel to the sagittal axis. While Verwey et al. (2002) stimulated SMA at the FCz location, which is 10% of the distance between inion and nasion (i.e. about 4 cm anterior to Cz), we here followed the recently more often reported location for the stimulation of SMAproper (Lefaucheur et al. 2020).

The stimulation intensity for cTBS was defined as 80% of the individual’s active motor threshold (AMT) (Huang et al. 2005). Stimulation intensity for rTMS was defined as 90% of the individual’s resting motor threshold (RMT) (Ziemann et al. 1998). While Verwey et al. (2002) determined the motor threshold as the intensity that showed movement of the thumb, wrist or any finger in 5 out of 10 trials (Pridmore et al. 1998), we here used motor threshold determination using electromyographic (EMG) activity. In order to determine RMT and AMT, the cortical “motor hotspot” of the musculus abductor digiti minimi (ADM) was determined for each participant using EMG (De Gennaro et al. 2003; Sohn et al. 2004). Bipolar electrodes were attached in a belly-tendon montage to the right ADM. Subsequently the location of the left motor cortex ADM representation was searched in steps of 1 cm starting at Cz until the coil position which resulted in the largest motor evoked potential (MEP) amplitude with a given medium TMS intensity was identified by the EMG. For this exploration, the figure-of-eight TMS coil was used while producing single pulses in five second intervals. First, the hotspot (the coil position over the primary motor area that produces the largest MEP in the right ADM with a given medium TMS intensity) was identified with TMS. Then, the stimulation intensity was adjusted to evoke MEPs with a peak-to-peak amplitude of ∼ 1 mV. During MEP recordings of the ADM, the figure of eight coil was positioned over the predefined cortical representation of ADM with the handle pointing backwards, and 45 degrees from midline in a lateral medial direction. Following this step, RMT and AMT were obtained.

RMT, used for determining rTMS intensity, was determined with the TMS Motor Threshold Assessment Tool (MTAT 2.0, http://www.clinicalresearcher.org/software.htm). The software proposes various TMS-pulse intensities to apply on the motor hotspot at the participants’ head. The software measures the intensities of the EMG responses to the given pulses and estimates the 95%-confidence interval for the resting motor threshold based on the collected data.

AMT was used to determine cTBS intensity. The AMT was determined by applying single-pulse biphasic TMS to the predefined cortical representation of the ADM muscle and recorded MEP with EMG electrodes. The TMS coil was again positioned 45 degrees from midline in a lateral to medial direction. The lowest TMS pulse intensity that evoked at least 3 MEPs of an amplitude of ∼ 200–300 μV via 6 TMS pulses during moderate tonic contraction of the ADM (∼ 20% of the EMG amplitude accomplished by maximal contraction of that muscle) was defined as the individual AMT.

**Fig. 1:**
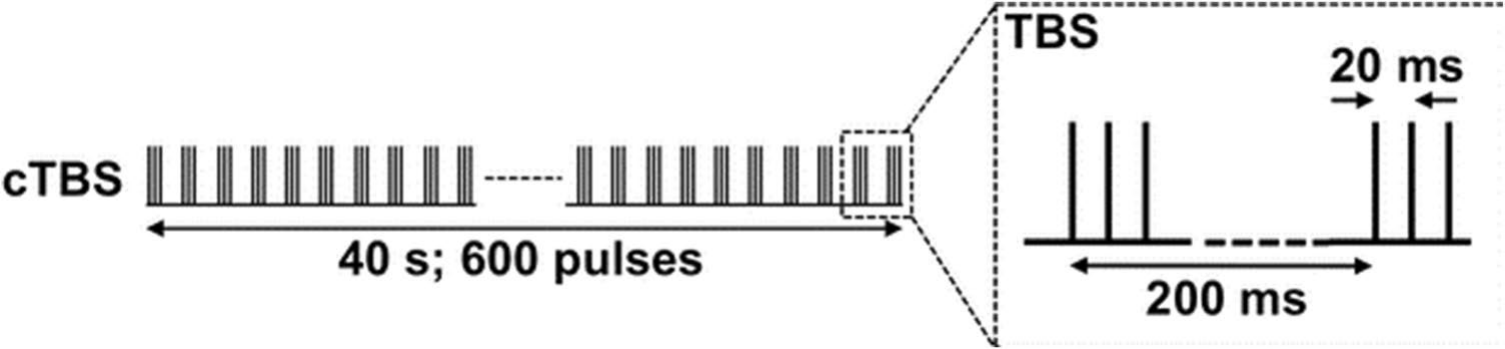
Graphical representation of the course of the continuous theta burst (cTBS) protocol showing the build up of the inter-burst interval (5 Hz; 200 ms) and intra-burst stimuli (50 Hz; 20 ms). Based on Wu et al. (2018)

cTBS involved 40 s stimulation yielding 600 magnetic pulses in total (see Fig. 1 for details). The rTMS protocol involved 1 Hz pulse administration for 20 min, which resulted in 1200 pulses. No adverse events occurred during the application of the non-invasive brain stimulation procedures.

### Procedure

When the participants arrived at the institute, they received an oral explanation of the study procedure. They then received a written description of the course of events in the study and signed the informed consent. Subsequently, participants were medically examined, as described above. They were asked to verbally reproduce the sequences that were given to them several days before they came to the lab. If they were not able to reproduce the sequences four times without an error, they received 15 additional minutes for learning and were retested. This appeared necessary for only one participant.

The next step was determining the individual stimulation intensity. The participants were seated in a chair designed for TMS application and their head was fixated. The motor hotspot was marked on each participant’s head. Then, depending of the participant’s group RMT or AMT was determined. The location of the SMA was determined by identifying Cz and marking the spot of SMA three centimeters anterior to Cz with a waterproof marker.

**Fig. 2:**
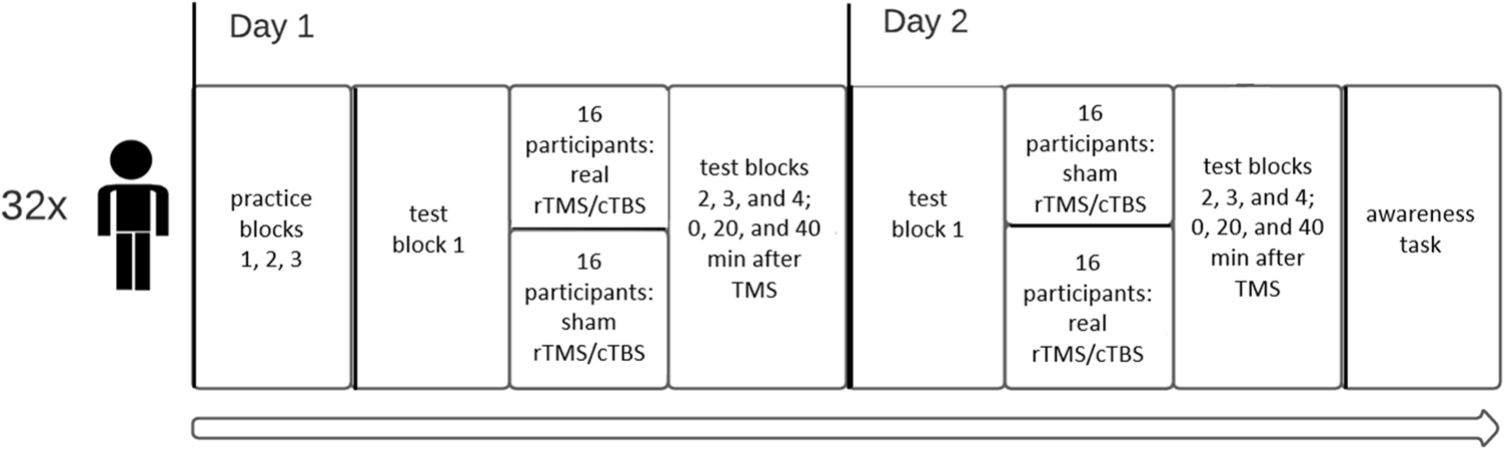
Procedure of the experiment excluding preparatory steps (sequence learning, medical examination, determination of AMT and RMT). On Day 1, 8 participants received real 1-Hz rTMS, 8 participants received sham 1-Hz rTMS, 8 participants received real cTBS, and 8 participants received sham cTBS. Test block one was used for baseline performance measurement. On Day 2, actual and sham stimulation were reversed for each participant. The post-TMS test blocks were performed 0, 20 and 40 min after completion of the stimulation block

Next, the lights were dimmed and the participants were seated in front of the experimental setup, the task instructions were given, and as shown in Fig. 2 the participants started practicing the sequences by pressing the keys in response to the sequence-specific stimulus. The little, ring, middle and index finger of the left hand were used to press the c, v, b and n keys, respectively. Participants practiced each of the two sequences 210 times in randomized order and divided across three 140-trial practice blocks. Each block involved a 20 s break halfway through and was followed by a 5 min pause. In both situations the screen showed a second counter going back to 0. At the end of the 20 s break the experiment automatically continued. After the 5 min break the experimenter entered the room and started the next block, which allowed him to regularly interact with the participant.

The first test block served as baseline for the subsequent test blocks and was followed by the actual or sham stimulation period. This involved fixating the head of the participant and positioning the coil with the coil holder on the skull over the SMA in the orientation described before. The stimulation took place in the chair the task was performed to avoid relocating the participant. The order of real and sham stimulation was counterbalanced across days in the cTBS and rTMS groups. Directly after the stimulation, the participants performed Test Block 2. Test Blocks 3 and 4 were performed 20 and 40 min after the end of TMS. The test blocks involved the same two DSP sequences as the practice blocks but a test block contained only 40 trials, again with the two sequences in random order.

After the four test blocks on day 1 had been finished the participants were thanked for their cooperation and were instructed about the experimental session on day 2. This included desisting from washing one’s hair to preserve the marks on the head that indicated the location of the motor hotspot and the SMA. The AMT or RMT were determined again at the beginning of day 2 using the “motor hotspot” mark. Next, the same test procedure was used as on day 1, including the first baseline test block, the required stimulation, and test blocks 2, 3 and 4. Participants stated that they tolerated the stimulation preceding the test phases well. After completion of the test blocks on day 2 the participants performed the awareness assessment task on the experimental computer. All experimental procedures took place under COVID-19-related safety measures.

### Data analysis

Mean RT for every participant, sequence and key press was calculated for each practice and test block. The first response time involved the time to react to the imperative stimulus and produce the first response. For the second and later responses RT involved the time between onset of two successive responses. Sequences containing an error were aborted and excluded from the RT analyses. Boxplot visualization showed no outliers. A mixed ANOVA was used to analyze RTs and arcsine transformed error proportions (Winer et al. 1991). In line with other DSP studies, we used Key (i.e., sequence positions 1 to 6) as independent variable in order to also assess potential differences of our manipulations of the first and later responses in the sequence. Data preparation and cleaning was done using E-Prime 2.0-DataAid, R and Microsoft Excel. Greenhouse–Geisser corrected p’s were used when sphericity assumptions were violated.

## Results

### Practice phase

A mixed ANOVA on RTs was carried out with TMS Group (2: rTMS vs. cTBS) and StimOrder Group (2: RS-Group/Day 1 Real Stimulation vs. SR-Group/Day 1 Sham) as between-subject variables. Block (3 practice blocks), Structure (2: 1 × 6 vs. 2 × 3 sequence), and Key (6) were within-subject variables. As expected, Block showed a significant main effect indicating improvement with practice, *F*(2,56) = 177.35, *p* < 0.01, *η*_*p*_^*2*^ = 0.86. The small difference between Blocks 2 and 3 (325 and 309 ms) relative to Block 1 (435 ms) suggests that participants were approaching the performance asymptote by the end of practice. Overall, 1 × 6 sequences were executed slower than 2 × 3 sequences, F(1,28) = 41.82, p < 0.001, *η*_*p*_^*2*^ = 0.60, but a Block x Structure interaction showed that this disadvantage for 1 × 6 reduced in later blocks, from 72 ms in Block 1 to 37 ms in both Blocks 2 and 3, *F*(2,56) = 12.06, *p* < 0.001, *η*_*p*_^*2*^ = 0.30.

The main effect of Key showed that RTs differed as a function of sequence position, *F*(5,140) = 209.74, *p* < 0.01, *η*_*p*_^*2*^ = 0.88. This could be attributed to the typically slow R_1_ (815 ms vs. R_23456_: 265 ms). Still, like in Verwey et al. (2002), segmentation was indicated by the slow R_4_ compared with *R*_2356_, (326 ms. vs. 250 ms), *F*(1,28) = 24.81, *p* < 0.001, *η*_*p*_^*2*^ = 0.47, and this was significant also for the 1 × 6 and 2 × 3 sequences separately, Fs(1,28) > 19.92, ps < 0.001, *η*_*p*_^*2*^s > 0.42. R_56_ were executed 27 ms faster than *R*_23_ (236 ms vs. 263 ms), *F*(1,28) = 40.63, *p* < 0.0001, *η*_*p*_^*2*^ = 0.59, and this too was the case for the 1 × 6 and 2 × 3 sequences individually, Fs(1,28) > 23.0, ps < 0.001, *η*_*p*_^*2*^s > 0.45.

A priori group differences were not observed between the two TMS-Groups, but they did occur for the two stimulation-order groups. The SR group appeared faster than the RS group even before TMS, especially so in 2 × 3 and this was caused by a faster *R*_123_ in the SR group. This was indicated by a marginally significant StimOrder-Group main effect (SR: 330 ms vs. RS: 383 ms), *F*(1,28) = 3.19, *p* = 0.08, *η*_*p*_^*2*^ = 0.10, and a StimOrder-Group x Structure x Key interaction, *F*(5,140) = 2.70, p = 0.05, *η*_*p*_^*2*^ = 0.09, that was superseded by StimOrder-Group x Structure, *F*(1,28) = 8.60, *p* = 0.007, *η*_*p*_^*2*^ = 0.23, and StimOrder-Group x Key interactions, *F*(5,140) = 4.34, *p* = 0.027, *η*_*p*_^*2*^ = 0.13.

An ANOVA with the same design on arcsine transformed errors showed that errors reduced from 1.9% per key press in Block 1 to 1.0% in both Blocks 2 and 3, *F*(2,56) = 31.29, *p* < 0.001, *η*_*p*_^*2*^ = 0.53. The higher error rate in Block 1 was caused mostly by the 1 × 6 sequence, but this disadvantage reduced to about the level of the 2 × 3 sequence in Blocks 2 and 3, *F*(2,56) = 7.10, *p* = 0.01, *η*_*p*_^*2*^ = 0.20. A main effect of Key showed that error rate was highest at *R*_1_ (2.2%), and was lower at later sequence positions (below 1.2% except 1.6% at R_5_), *F*(5,140) = 15.15, *p* < 0.0001, *η*_*p*_^*2*^ = 0.35. A Key x Block interaction, *F*(10,280) = 6.68, *p* < 0.02, *η*_*p*_^*2*^ = 0.19, showed that errors reduced from Block 1 to Block 2 and 3 for *R*_1234_, and not for *R*_56_. The other main effects and interactions were not significant.

### Test phase

RTs were analyzed with a mixed ANOVA with TMS-Group (2: rTMS vs. cTBS) and StimOrder-Group (2: RS vs. SR group) as between-subject factors, and Stimulation (2: Real vs. Sham), Structure (2: 1 × 6 and 2 × 3), Delay (4: Baseline, 0, 20, and 40 min) and Key (6) as within-subject factors. This analysis confirmed that the RS group was generally slower than the SR group (319 ms vs. 264 ms), *F*(1,28) = 5.35, *p* = 0.03, *η*_*p*_^*2*^ = 0.16, even in the first test blocks that preceded stimulation (327 ms vs. 275 ms), *F*(1,28) = 3.73, *p* = 0.06, *η*_*p*_^*2*^ = 0.12.

**Fig. 3:**
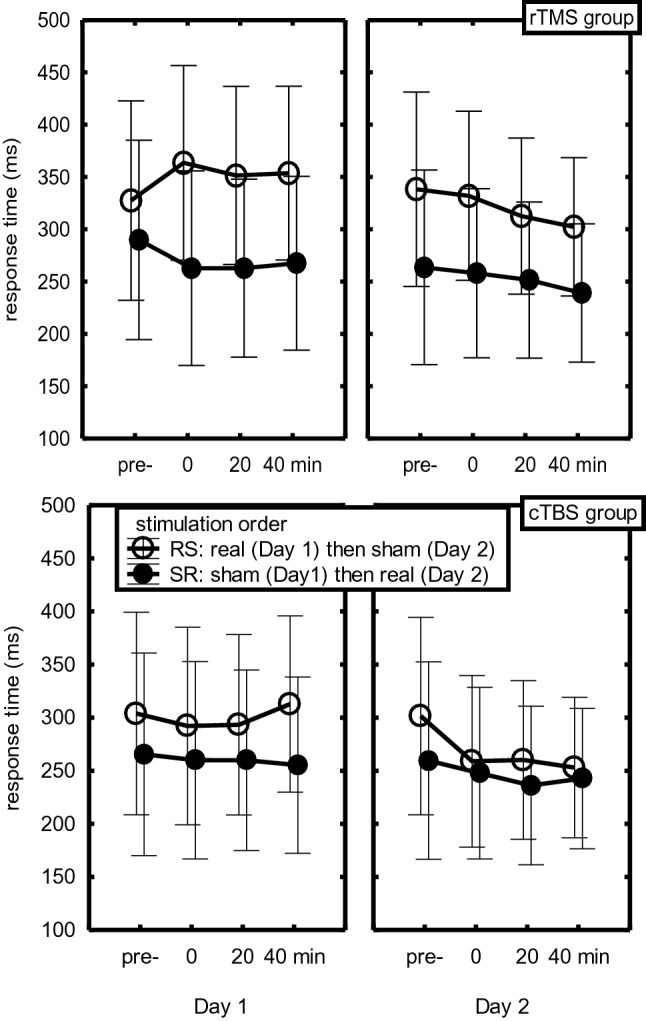
Response time before and 0, 20 and 40 min after completion of rTMS and cTBS administration in the real and sham stimulation conditions on days 1 and 2

The delay main effect reflected general improvement across the four successive test blocks (301, 292, 287, 286 ms), *F*(3,84) = 5.84, *p* = 0.02, *η*_*p*_^*2*^ = 0.17. However, in line with slowing by real stimulation, a Stimulation x Delay interaction showed that this improvement was larger in the sham than in the real stimulation condition, *F*(3,84) = 5.15, *p* = 0.03, *η*_*p*_^*2*^ = 0.16. Further analyses revealed that slowing by rTMS stimulation, relative to pre-stimulation, was significant for each of the three delays, Fs(1,28) > 4.91, ps < 0.04, *η*_*p*_^*2*^s > 0.15. It should be noted that the RT difference between real and sham stimulation in the pre-TMS block that is visible in Fig. 3 was not significant, *F*(1,28) = 2.69, *p* = 0.11. For the cTBS group, the slowing by real stimulation relative to pre-stimulation was significant after 40 min (28 ms slowing), *F*(1,28) = 4.33, *p* < 0.05, *η*_*p*_^*2*^ = 0.13, but not after 0 and 20 min (14 ms and 7 ms slowing, respectively), Fs(1,28) < 1.44, ps > 0.24. Hence, across the RS and SR groups rTMS slowed sequence execution after 0, 20 and 40 min. Instead, cTBS slowed sequence execution only after 40 min.

Importantly, a StimOrder-Group x Stimulation x Delay interaction confirmed that the effect of real stimulation, relative to sham stimulation, differed for participants actually stimulated on day 1 (the RS group) and on day 2 (SR group), *F*(3,84) = 7.27, *p* = 0.01, *η*_*p*_^*2*^ = 0.21. This StimOrder-Group x Stimulation x Delay interaction was significant also for both TMS groups separately, Fs(3,84) > 3.68, ps > 0.02, *η*_*p*_*s*^*2*^ > 0.12. Figure 3 shows the causes of these interactions. Apart from confirming that the SR group was generally faster than the RS group, this figure shows that for the RS group real stimulation with rTMS on day 1 increased RTs at all delays relative to pre-TMS (top left frame), whereas after sham rTMS on day 2, RTs showed the normal practice effect across test blocks (top right frame). The cTBS group also showed that slowing by TMS occurred only on day 1 where it was, as reported above, limited to the 40 min delay. On day 2, sham cTBS was followed by the normal RTs improvement across test blocks. Planned comparisons confirmed these findings in that for the RS group the effect of delay was different after real stimulation on day 1 and sham stimulation on day 2 for both the rTMS group, *F*(3,83) = 6.76, *p* < 0.001, *η*_*p*_^*2*^ = 0.19, and the cTBS group, *F*(3,84) = 5.32, *p* = 0.002, *η*_*p*_^*2*^ = 0.16. In contrast, planned comparisons for the SR group showed that the effect of delay did not differ after sham TMS on day 1 and after real stimulation on day 2 for the rTMS group, *F*(3,84) = 0.90, *p* = 0.44, and neither for the cTBS group, *F*(3,84) = 0.70, *p* = 0.55. Further planned comparisons showed that this differential effect of delay for the RS and SR groups was significant when tested separately for the rTMS group, *F*(3,84) = 3.81, *p* = 0.013, *η*_*p*_^*2*^ = 0.12, and the cTBS group, *F*(3,84) = 3.68, *p* = 0.015, *η*_*p*_^*2*^ = 0.12. Therefore, the RT increase after real TMS was significant on day 1 and not on day 2 and this was the case for both the rTMS and the cTBS group.

To determine whether offline consolidation had improved performance on day 2 before administration of TMS, we compared for the SR group RTs in the pre-stimulation test blocks on days 1 and 2 (Fig. 3). This showed no significant RT decrease after 24 h, *F*(1,28) = 2.54, *p* = 0.12.

**Fig. 4:**
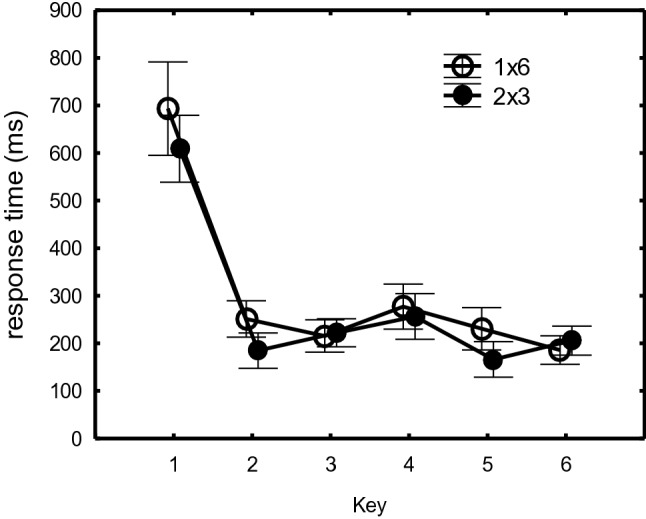
Response times in the 1 × 6 and 2 × 3 sequences as a function of sequence position collapsed across the rTMS and cTBS, and the RS and SR groups

The ANOVA further showed the typical key effect, indicating especially the slow *R*_1_ relative to *R*_23456_ (651 ms vs. 220 ms), *F*(5,140) = 198.42, *p* < 0.001, *η*_*p*_^*2*^ = 0.88. However, across all conditions, it was caused also by a relatively slow *R*_4_ relative to *R*_2356_ (267 ms vs. 208 ms) which effect confirms the development of segmentation, *F*(1,28) = 26.62, *p* < 0.0001, *η*_*p*_^*2*^ = 0.49 (Fig. 4). Like during practice, the first segment (*R*_23_) was executed 20 ms slower than the second segment (*R*_56_) (218 ms vs. 198 ms), *F*(1,28) = 41.41, *p* < 0.001, *η*_*p*_^*2*^ = 0.60. The Structure main effect showed that 1 × 6 sequences were still carried out slower than 2 × 3 sequences (309 vs. 274 ms), *F*(1,28) = 25.77, *p* < 0.001, *η*_*p*_^*2*^ = 0.48. Importantly, there were no significant interactions with Key that would suggest that stimulation had different effects on the 6 key positions.

**Fig. 5:**
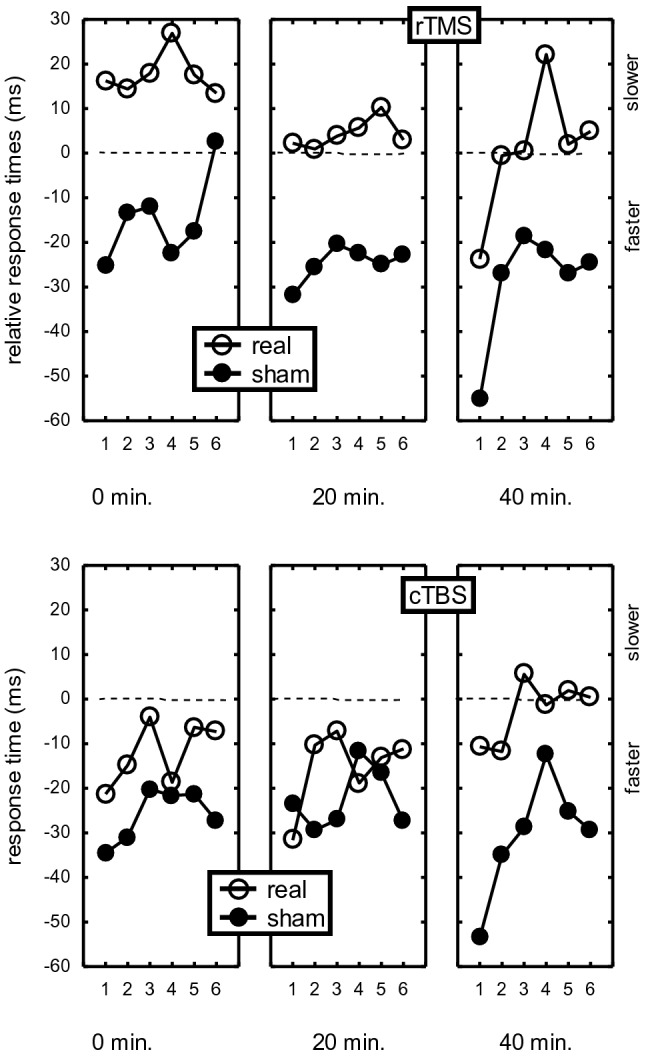
Response time 0, 20 and 40 min after stimulation relative to the block before rTMS and cTBS stimulation as a function of real and sham stimulation for each key position. ‘Faster’ indicates shorter relative RTs after than before TMS, which is probably caused by improvement across test blocks, while ‘slower’ indicates longer RTs after stimulation which can be accounted for by TMS

Just like in Verwey et al. (2002), we determined the TMS effect for each individual key by computing RTs in the 0, 20, and 40 post-stimulation tests relative to the pre-TMS condition. This involved subtracting from RT at each sequence position in the three test blocks following stimulation, the corresponding RT assessed in the block that preceded stimulation. We analyzed these *relative RTs* with a similar ANOVA as the absolute RTs above. Fig. 5 shows the effects of stimulation in both TMS groups (notice that these effects were about twice as large for the RS group alone). The ANOVA confirmed by way of a stimulation main effect that relative RTs were not slower in the three post-sham stimulation test blocks (i.e., than in the pre-TMS block), while there was a clear RT increase after real stimulation (− 1 vs. − 25 ms, respectively), *F*(1,28) = 8.86, *p* = 0.006, *η*_*p*_^*2*^ = 0.24. The Stimulation x StimOrder-Group interaction confirmed that the slowing effect of real over sham TMS was larger for the RS group with actual stimulation on day 1 (actual rTMS: 14 ms vs. sham: − 36 ms) than in the SR group with actual stimulation on day 2 (actual rTMS: − 16 ms vs. sham: − 13 ms), *F*(1,28) = 11.09, *p* = 0.002, *η*_*p*_^*2*^ = 0.28. Higher order interactions that included TMS-group and delay did not reach statistical significance. Again, none of the interactions involving key and stimulation were significant indicating that the slowing effect of real rTMS and real cTBS concerned all six responses in the sequence.

The same ANOVA as with absolute RTs was used to analyze arcsine transformed error proportions. It showed by way of a Delay main effect that error rate was lowest before stimulation and was higher after stimulation (0.6, 0.9, 0.9, 0.8%, per key, respectively), *F*(3,64) = 2.90, *p* = 0.04, *η*_*p*_^*2*^ = 0.12. More errors were made in the 1 × 6 than in the 2 × 3 sequences (1.0% vs. 0.7% per key, respectively), *F*(1,28) = 4.33, *p* = 0.05, *η*_*p*_^*2*^ = 0.13. Error rate was relatively low for *R*_2_ and *R*_6_ and higher for *R*_5_ (0.8, 0.4, 0.8, 0.9, 1.6, 0.4%, respectively), *F*(5,140) = 9.25, *p* < 0.001, *η*_*p*_^*2*^ = 0.25, and this high *R*_5_ error rate occurred only in the 1 × 6 sequence, *F*(5,140) = 6.64, *p* < 0.001, *η*_*p*_^*2*^ = 0.19.

In summary, the results basically replicate the earlier findings with rTMS reported in Verwey et al. (2002) in that across both days, slowing was observed at all sequence positions and amounted to about 30 ms. However, this time rTMS slowed sequence execution also after 0 and 40 min. cTBS induced significant slowing by 28 ms, but this was seen only 40 min after stimulation. Further scrutiny showed that after rTMS and cTBS there was no slowing after stimulation on day 2 while on day 1 slowing was in fact about twice as large as the above reported values of 30 ms and 28 ms. The results further confirmed typical effects of key position like a slow *R*_1_ and *R*_4_, and a slower first segment (*R*_23_) than a second segment (*R*_56_), and that 1 × 6 was executed slower than 2 × 3, but R_1_ and R_4_ were not slowed more by TMS than the other responses.

### Awareness task

The results of the awareness task at the end of day 2 showed that three of the 16 rTMS and three of the 16 cTBS participants reproduced their two sequences correctly in both the spatial and verbal tests. This is surprisingly poor given that participants started off executing their sequences on day 1 on the basis of two verbally learned letter series.

A nonparametric mixed 2 (TMS-Group: cTBS vs. rTMS) × 2 (Task: Spatial versus Verbal) × 2 (Structure: 1 × 6 versus 2 × 3) ANOVA was carried out with TMS-Group as between-subject variable on the mean numbers of correct responses per sequential position. This involved an F1–LD–F2 design using the nparLD package (Noguchi et al. 2012) in R Studio (version 1.3.1093, R-Core_Team 2020)^2^. Two further nonparametric ANOVAs, one for Spatial and one for Verbal Test data, used stimulation-order instead of Task as independent variable in an F2–LD–F1 design. The only significant effect was that performance in the Verbal Test was poorer for the 1 × 6 than for the 2 × 3 sequence (4.1 versus 5.0 correct of 6, respectively), while there was little difference for the 2 sequences in the Spatial test (5.3 vs. 5.4, respectively), ATS(1) = 3.75, *p* = 0.05. This interaction was responsible also for the main effects of Task, ATS (1) = 5.63, *p* = 0.02, and Structure, ATS (1) = 4.46, *p* = 0.03. Performance on the awareness task was not different in the rTMS and cTBS groups, ATS (1) = 0.29, *p* = 0.59, and neither for the SR and RS groups, ATS (1) < 0.54, ps > 0.46. Correlations between the number of correct responses in the awareness test and total execution times in the three practice blocks and in the test blocks on days 1 and 2 were never significant (ps > 0.16).

Table 1 indicates that the strategies participants claimed to have used when performing the spatial and the verbal tests, and how certain they had been about their answers. These results show that participants relied mostly on the letter series they had learned earlier and on imagining to execute the sequences. They also indicate that the high certainty of at least 7 of the 32 participants (13 'very certain' participants minus 6 participants with correct explicit knowledge) was unjustified. The participants seemed not always able to estimate the reliability of their awareness. Finally, out of the 16 rTMS participants, 8 correctly identified the real stimulation session (‘today’ vs. ‘yesterday’) and 8 did this incorrectly. With cTBS, 8 were 6 were correct and 10 incorrect. This indicates that participants had very little awareness of the true stimulation session.

**Table 1:** Numbers of participants showing full explicit knowledge, the strategy they said to have used to perform the spatial and verbal awareness tests, and how certain they had been about their awareness

	Spatial test	Verbal test
Nr. of participants with 2 correct sequences (of 32)	6	6
Letters on keys	10	13
Stimulus/key locations	7	5
Tapping on table top	2	0
Tapping in the mind	11	8
no idea	2	6
Subjective certainty (from 'very certain' to 'very uncertain', resp.)	13 9 7 3	13 7 4 8

## Discussion

A prime goal of the present study was to test whether susceptibility of the SMAproper to TMS reduces due to offline consolidation. A second goal was whether specifically targeting SMAproper would slow all responses of a practiced DSP sequence. Lastly, the experiment was used to investigate whether the 40 s cTBS protocol would have the same behavioral effects 0, 20 and 40 min after stimulation as the 20 min rTMS protocol.

### Consolidation

As predicted, both rTMS and cTBS slowed execution of the DSP sequences when administered immediately after practice on day 1, while this was not observed on day 2. This confirms our suspicion that the slowing of responses by rTMS reported in Verwey et al. (2002) was based solely on the results of the test session on day 1. This corroborates that offline consolidation following practice stabilizes learning and makes it robust against interference of SMAproper by TMS. Offline consolidation has recently been argued to result from repeatedly preparing keying sequences in short term memory (Verwey et al. 2021). We did not observe indications that stimulation of SMA on day 1 hampered the ensuing offline consolidation (Kim and Wright 2020).

The present results suggest that SMAproper is involved in the application and consolidation of implicit, and not of explicit, sequence knowledge. Even though participants initially required explicit verbal sequence knowledge to learn executing the sequences, at the end of day 2 performance of the participants on the awareness task was unexpectedly poor. Only 6 of 32 participants showed perfect reproduction of the two sequences in the awareness test. The amount of directly available explicit sequence knowledge was probably even less as earlier research showed that during awareness tests participants tend to reconstruct their sequences using implicit knowledge (Verwey 2015; Verwey et al. 2015). In addition, explicit knowledge has been shown to contribute little to rapidly executed DSP sequences (e.g., Cleeremans and Sarrazin 2007; Verwey 2015; Verwey and Wright 2014). Finally, there is no reason from other studies to expect SMA to be involved in explicit verbal knowledge. In line with these arguments, correlations were small and insignificant between awareness at the end of day 2 and execution rate during practice individual test blocks.

### The role of SMAproper

The rTMS condition replicated most of the effects of the earlier study (Verwey et al. 2002). We also found the spontaneous development of a relatively slow fourth response that is taken as indication for segmentation (Abrahamse et al. 2013; Verwey et al. 2009). Still, in contrast to when preSMA was stimulated (Kennerley et al. 2004; Ruitenberg et al. 2014), the present stimulation of the SMAproper did not slow the first and fourth responses more than the other key presses. These results are consistent with the notion that TMS affected the role of SMAproper in triggering responses in M1 but, given the proven anatomical connectivity between SMAproper and M1 (Arai et al. 2012), the behavioral effects of TMS may have emerged also from an enduring effect on the excitability of M1.

When averaged across both days and the three test delays, the present rTMS-induced slowing of individual keypresses by about 30 ms seems to exceed the 19 ms slowing in Verwey et al. (2002). In addition, in the present study rTMS slowed the responses immediately after stimulation too. These stronger effects of rTMS in the present study may have resulted from stimulating 1 cm more posteriorly which affected SMAproper more, but we cannot exclude that they were caused by the more sophisticated motor threshold assessment procedure yielding another stimulation intensity.

### The TMS protocols

The results suggest that 40 s cTBS cannot always be used to replace 20 min rTMS stimulation. While offline consolidation and slowing 40 min after TMS were similar in the cTBS and rTMS conditions, cTBS did not show sequence slowing 0 and 20 min after stimulation like rTMS did.

The delays after which the TMS effects were assessed were relative to the end of the cTBS and rTMS stimulation periods because we were interested in the possibility to replace 20 min of rTMS by 40 s of cTBS. However, even if we compare the RT effects relative to the start of stimulation, the significant slowing by cTBS did not occur yet after 20 min, while it did occur 20 min after the start of rTMS (i.e., 0 min after rTMS completion). Therefore, even relative to the onset of the stimulation period the slowing effect of cTBS developed more slowly than of rTMS. Still, given the known variability of TMS effects this difference may have resulted from the limited power of the present study and future research should determine whether these differences between rTMS and cTBS are robust.

## Conclusions

The present results replicated most of the results of the earlier DSP study that used rTMS to stimulate SMA (Verwey et al. 2002). They add three new conclusions. First, they confirm that practice is followed by offline consolidation in the 24 hours following practice that makes sequence execution robust against stimulation of SMAproper with rTMS and cTBS. Second, SMAproper is involved in triggering each response in a familiar DSP sequence using implicit sequence knowledge. Third, the effects of 40 s cTBS differed from those of 20 min rTMS in that cTBS slowed sequence execution only 40 min after stimulation on the day of practice, whereas rTMS slowed sequence execution 0, 20 and 40 min after stimulation on that day.

## Data Availability

The E-Prime source codes and data are available on the site of the Open Science Framework (https://osf.io/m8hdk/?view_only=f72ce908ee984a898811d8772bd7694b).

## Footnotes

1 As the responses after the first in the typical DSP paradigm are eventually not given in reaction to a stimulus, studies using the DSP task commonly use RT as abbreviation of *response time* rather than of reaction time (Verwey 1996).

2 More than 2 between- and 2 within-subject independent variables are not possible with nparLD.
